# Real-World Use of Biologic Therapy in Elderly Patients with Moderate-to-Severe Psoriasis: A Retrospective Single-Center Study

**DOI:** 10.3390/medicina62050911

**Published:** 2026-05-08

**Authors:** Vanda Bondare-Ansberga, Aleksandra Grigorjeva

**Affiliations:** 1 Department of Dermatology and Venereology, Riga Stradins University, Baznicas Street 18, LV-1010 Riga, Latvia; 2 Riga 1st Hospital, Bruninieku Street 5, LV-1001 Riga, Latvia

**Keywords:** psoriasis, elderly, biologic therapy

## Abstract

*Background and Objectives*: Psoriasis patients aged over 65 years, especially those with chronic comorbidities, constitute an increasingly important patient population. In this group, the selection of therapy, along with its efficacy and safety, is of relevance. *Materials and Methods*: A single-center retrospective analysis of clinical records of patients aged ≥ 65 years with moderate to severe plaque psoriasis, treated with biologic therapies. The study covered the period 2019–2025. *Results*: A total of 38 patients were included. Biologic switching occurred in 15 patients (39.5%). The primary reason for switching was loss of efficacy (86.7%), while adverse events accounted for 13.3% of cases. The mean duration from initiation of adalimumab (the mandatory first-line biologic therapy) to switching was 325 ± 191 days. Baseline PASI did not differ significantly between groups (*p* = 0.139). Psoriatic arthritis was significantly less frequent among patients who switched therapy (13.3% vs. 56.5%, *p* = 0.008). No significant associations were observed between switching and comorbidities or overall comorbidity burden (*p* > 0.05). Clinically relevant adverse events included infections, cardiovascular events, malignancies, and autoimmune laboratory abnormalities. *Conclusions*: Biologic therapy in elderly psoriasis patients appears to be generally well tolerated, based on descriptive real-world data. Treatment switching was primarily driven by loss of efficacy rather than comorbidities.

## 1. Introduction

Psoriasis is a chronic, immune-mediated, inflammatory, noncommunicable disease. The reported prevalence of psoriasis in countries ranges between 0.09% and 11.4% [1]. The age-specific prevalence rate in 2017 showed psoriasis peak between 60 and 70 years of age. Psoriasis burden was greatest in countries with high income. A positive linear relationship has been observed between psoriasis prevalence and comorbidities, including cardiovascular disease, mental health disorders, type 2 diabetes mellitus, stroke, lymphoma, nonmelanoma skin cancer, and inflammatory bowel disease across all countries in 2017 [2].

Individuals living with psoriasis are more likely to develop multiple long-term conditions at an earlier age compared with those without psoriasis [3]. Therefore, the management of psoriasis in elderly patients can be particularly challenging [4]. Patients of advanced age are commonly underrepresented in clinical trials and are often considered to have an increased risk of adverse events [5].

The World Health Organization (WHO) often uses ≥60 years as a definition of “older” or “elderly” in global health contexts [6]. According to the United Nations, older persons are defined as persons aged 65 years or over [7]. Elderly patients were defined as those aged 65 years or older, in accordance with International Council for Harmonization (ICH) E7 guidelines [8]. In this study, elderly patients are defined as those aged 65 years or older, consistent with international regulatory guidance for geriatric populations (ICH E7).

According to the summary of product characteristics, no dose adjustment is required for biologic therapies in elderly patients; however, caution is advised due to an increased risk of infections and the limited representation of this population in clinical trials. Available evidence suggests that the safety and efficacy profiles of newer biologics, particularly IL-17 and IL-23 inhibitors, are generally comparable between elderly and younger patients [9,10,11,12,13]. There is limited information in patients aged ≥ 65 years and very limited information in patients aged ≥ 75 years [11,12].

Biologics in Latvia are indicated for the treatment of moderate to severe chronic psoriasis in patients for whom conventional systemic therapy with cyclosporin A, synthetic retinoids, methotrexate and phototherapy has been ineffective, contraindicated or poorly tolerated [14]. According to the Latvian National Health Service List of Diagnoses and Reimbursable Medicines/Medical Devices, adalimumab should be selected as the first biologic for all psoriasis patients who meet the criteria for initiating biologic therapy. ustekinumab, secukinumab, guselkumab, or risankizumab may be prescribed by a dermatologist, venereologist in accordance with the decision of a dermatology–venereology consultation board at Pauls Stradiņš Clinical University Hospital or Riga East Clinical University Hospital, for patients with moderate to severe chronic psoriasis (severity index—PASI ≥ 10; body surface area involvement—BSA ≥ 10%) who have not responded to previous treatment with Adalimumab [15].

Despite the publication of multiple real-world studies on biologic therapy in elderly psoriasis patients [16,17,18,19,20,21], there remains an unmet need for further research in this population. In this context, although this is a single-center study, the Dermatology and STI Clinic of Riga 1st Hospital functions as a specialized psoriasis treatment center, representing a substantial proportion of patients receiving biologic therapy in Latvia. However, due to the absence of a national psoriasis registry, the exact representativeness of this cohort cannot be precisely determined.

The aim of this study was to describe treatment patterns, biologic switching, and safety of biologic therapy in elderly patients with psoriasis in a real-world clinical setting.

## 2. Materials and Methods

A retrospective single-center study was conducted at the dermatology clinic of Riga 1st Hospital. Medical records from 2019 to 2025 were reviewed. In total, 337 patient records were screened, of which 286 patients had received biologic therapy specifically for the treatment of psoriasis. Among these, 38 patients aged ≥ 65 years were identified and included in the present analysis.

Elderly patients, defined as those aged ≥ 65 years, were included in the detailed analysis. Clinical data were extracted from patient records, including demographic characteristics (age and sex), disease-related variables (PASI, BSA), and involvement of difficult-to-treat areas (scalp, nails, palms, soles, and genital region). Information on comorbidities and treatment history, including biologic therapy and switching patterns, was also collected. Biologic therapy in this study was initiated and managed exclusively based on dermatological indications, with the primary focus on cutaneous disease activity (e.g., PASI, BSA). Psoriatic arthritis was recorded as a comorbidity and was not used as a determinant of treatment switching. Treatment decisions, including switching, were made by dermatologists, and no switching was attributed solely to psoriatic arthritis progression. Rheumatological indications were managed through a separate clinical pathway and were not included in the present analysis.

Comorbidities were categorized as follows: thyroid disease included autoimmune thyroiditis and thyroid nodules classified as TI-RADS III or higher. Dyslipidemia was recorded based on documented diagnoses in patient medical records and may therefore be underestimated. Diabetes comprised both type 1 and type 2 diabetes mellitus, while obesity was defined as a body mass index (BMI) ≥ 30 kg/m^2^. Cardiovascular diseases encompass chronic venous insufficiency, stable angina, heart failure, and cardiac arrhythmias. Lung disease refers to chronic obstructive pulmonary disease (COPD) and bronchial asthma. Kidney disease was defined as chronic kidney disease or chronic renal parenchymal damage. Infection history covered hepatitis B virus (HBV) and hepatitis C virus (HCV) infections.

All included patient records contained sufficient clinical data for analysis.

The primary outcome of the study was biologic switching, analyzed as a binary variable (switch vs. no switch).

Statistical analysis was performed using IBM SPSS Statistics (version 31.0, IBM Corp., Armonk, NY, USA) and Microsoft Excel (version 16.103.3, Microsoft Corp., Redmond, WA, USA). Descriptive statistics were used to summarize patient characteristics. Continuous variables are presented as mean ± standard deviation (SD), while categorical variables are expressed as number (percentage). Comparisons between groups (patients who switched biologic therapy and those who did not) were performed using the independent-samples t-test for continuous variables and the chi-square test or Fisher’s exact test for categorical variables, as appropriate. A *p*-value of <0.05 was considered statistically significant.

Biologic switching was analyzed as a binary outcome due to the retrospective design. Switching was assessed over the entire available follow-up period for each patient, from initiation of biologic therapy until switching or last available clinical record.

As complete and consistent time-to-event data, including follow-up duration for all patients, were not available, time-to-event analyses (e.g., drug survival or Kaplan–Meier analysis) were not performed.

## 3. Results

### 3.1. Baseline Patient Characteristics

During the study period, 337 patient records were screened, of which 286 corresponded to patients treated with biologic therapy for psoriasis. Among these, 38 patients (13.3%) were aged ≥ 65 years and formed the study population for further analysis.

The mean age of the cohort was 70.7 ± 4.7 years, and 55.3% were female. Overall, patients had moderate-to-severe disease with a mean PASI of 19.3 ± 10.2 and BSA of 15.0 ± 19.1. Comorbidities were common, with a mean of 2.3 ± 1.5 comorbid conditions per patient.

Detailed baseline characteristics, including disease manifestations, treatment history, and comorbidities, are presented in Table 1.

### 3.2. Biologic Switching and Associated Factors

Biologic switching occurred in 15 patients (39.5%). The primary reason for switching was loss of efficacy, while a smaller proportion of patients switched therapy due to adverse events. Two patients required escalation to third-line biologic therapy.

Among patients who switched therapy, guselkumab and risankizumab were the most frequently used second-line biologics, followed by ustekinumab and secukinumab. Detailed switching patterns are presented in Table 2.

Among patients who switched, the mean duration of adalimumab treatment prior to switching was 325 ± 191 days (range: 74–841 days). As adalimumab was the first-line biologic therapy in all patients according to national reimbursement policy, treatment duration was assessed from initiation of adalimumab to switching, representing a consistent reference point across the cohort.

No significant differences were observed in baseline PASI between patients who switched therapy and those who did not. Psoriatic arthritis was significantly less frequent among patients who switched therapy; however, this finding should be interpreted as a descriptive association rather than a causal factor, as treatment decisions were based exclusively on dermatological disease activity. No statistically significant associations were found between biologic switching and comorbidities. Detailed univariate comparisons between groups are presented in Table 3.

### 3.3. Safety and Adverse Events

Treatment discontinuation due to adverse events or patient-related factors was observed in 4 of 38 patients (10.5%), all of whom were receiving adalimumab at the time.

Clinically relevant adverse events and treatment-related complications were observed predominantly during adalimumab therapy.

Infectious complications included one case of latent tuberculosis, which required temporary interruption of therapy, after which treatment was successfully resumed with risankizumab. Another patient discontinued therapy due to recurrent infections, including pneumonia.

Malignancies diagnosed during biologic therapy included renal cancer, leading to permanent discontinuation of adalimumab, and papillary thyroid carcinoma, for which total thyroidectomy was performed; in this case, biologic therapy (ustekinumab) was continued with oncologist approval.

Cardiovascular and systemic adverse reactions were also observed. One patient developed tachycardia and hypertension within two days of adalimumab initiation. Subsequent laboratory evaluation, performed after treatment discontinuation, revealed a positive antinuclear antibody (ANA) titer, and the patient declined further biologic therapy. Another patient developed exertional angina requiring coronary stent placement during biologic therapy.

Cutaneous reactions included a localized injection-site reaction with pruritus after the fourth adalimumab dose; histopathology demonstrated psoriatic changes, and treatment was subsequently switched to guselkumab.

In addition, one patient experienced disease progression with the development of new lesions during adalimumab therapy; although a switch to an alternative biologic agent was recommended, the patient declined treatment modification.

## 4. Discussion

Elderly patients with psoriasis represent a growing and clinically complex population, characterized by a high burden of comorbidities and frequent underrepresentation in randomized clinical trials, which limits the available evidence for optimal treatment strategies. Real-world studies are therefore essential to better understand treatment patterns, safety, and effectiveness in this population. In the present single-center retrospective study, biologic therapy appeared to have a favorable safety and effectiveness profile in patients aged ≥ 65 years, supporting its use in routine clinical practice.

The observed biologic switching rate of 39.5% is consistent with previously reported real-world evidence, where switching rates in psoriasis patients typically range between 30% and 50%, particularly in older populations [16,18,19,20]. In our cohort, switching was predominantly driven by loss of efficacy (86.7%), whereas adverse events accounted for a minority of cases. This finding aligns with existing literature suggesting that loss of efficacy, rather than safety concerns, is the primary driver of treatment modification in patients receiving biologic therapy [18,19,20]. The predominance of loss of efficacy may reflect the complex and heterogeneous immunopathogenesis of psoriasis, as well as age-related changes in immune function and pharmacodynamics that may influence treatment response in elderly patients. In addition, treatment selection in elderly patients is often influenced by factors such as comorbidities, polypharmacy, and potential drug interactions, which may further complicate therapeutic decision-making [4,21].

In Latvia, reimbursement policies require adalimumab as the first-line biologic therapy, which may influence treatment patterns and switching behavior. Subsequent biologic selection (e.g., ustekinumab, secukinumab, guselkumab, or risankizumab) is based on individual patient characteristics, including comorbidities, presence of psoriatic arthritis, and clinical judgment, as no strict hierarchy exists beyond first-line therapy.

Previous real-world registry data have also suggested that patient-related factors, including sex, may influence treatment outcomes. Female patients have been reported to demonstrate lower drug survival and higher rates of adverse events during biologic therapy [22]. Although sex-specific analyses were beyond the scope of the present study, such factors may contribute to variability in treatment persistence and should be considered when interpreting real-world treatment patterns.

Although psoriatic arthritis was less frequent among patients who switched biologic therapy, this observation should be interpreted with caution. In this study, treatment decisions were based exclusively on dermatological indications, and psoriatic arthritis was considered as a comorbidity rather than a driver of switching. Therefore, this finding represents a descriptive association rather than a causal relationship.

Comorbidities were highly prevalent in our cohort, with a mean of 2.3 comorbid conditions per patient. Despite this, no significant associations were identified between comorbidity burden and biologic switching. This finding is consistent with previous studies indicating that biologic therapies can be safely and effectively used in elderly patients with multiple comorbidities [4,18,20,21]. It also suggests that, in clinical practice, treatment decisions may be more strongly influenced by disease activity and therapeutic response rather than the presence of comorbid conditions alone. Nevertheless, careful patient selection and individualized risk assessment remain essential, particularly in patients with cardiovascular disease, malignancy, or chronic infections.

Additionally, the use of a standardized comorbidity index was not feasible, which limits the ability to systematically and quantitatively assess comorbidity burden.

The overall safety profile observed in this study was favorable, with clinically relevant adverse events occurring in 18.4% of patients and treatment discontinuation due to adverse events in only 10.5% of cases. These findings are in line with previous real-world studies demonstrating that biologic therapies are generally well tolerated in elderly populations [4,19,20]. The spectrum of adverse events observed—including infections, cardiovascular events, malignancies, and autoimmune phenomena—is consistent with known safety profiles of biologic agents. Notably, infectious complications and malignancies, although infrequent, underscore the importance of ongoing monitoring in this vulnerable population. These findings support the notion that, despite age-related vulnerability, biologic therapies maintain a favorable risk–benefit profile in elderly patients when appropriate monitoring is implemented.

Our findings further support the growing body of evidence indicating that newer biologic agents, particularly interleukin IL-17 and IL-23 inhibitors, demonstrate favorable safety profiles in elderly patients [4,18,19,20,21]. These therapies offer targeted mechanisms of action and may be associated with improved tolerability compared with traditional systemic treatments. However, data on patients aged ≥ 75 years remain limited, and further research is needed to better characterize long-term safety outcomes in this subgroup.

From a clinical perspective, the results of this study support the use of biologic therapy in elderly psoriasis patients, even in the presence of multiple comorbidities. Treatment decisions should prioritize efficacy while ensuring careful monitoring for adverse events. The relatively low rate of discontinuation due to safety concerns suggests that biologic therapies can be maintained in appropriately selected patients.

This study has several limitations. First, the relatively small sample size limits the statistical power and may result in unstable subgroup analyses. Second, the retrospective single-center design may introduce selection bias and limit generalizability. Although conducted in a specialized psoriasis treatment center, the absence of a national registry prevents precise assessment of representativeness.

Third, the analysis was limited to univariate methods without adjustment for potential confounders, which restricts the ability to identify independent predictors of biologic switching. Additionally, no correction for multiple comparisons was applied, which increases the risk of type I error and false-positive findings. Therefore, statistically significant results should be interpreted with caution. Fourth, although dates of biologic initiation and switching were available for a subset of patients, complete and consistent time-to-event data, including follow-up duration for patients without switching, were not available for all cases. Therefore, treatment persistence and drug survival could not be assessed using time-to-event methods such as Kaplan–Meier or Cox regression analysis. Importantly, the absence of time-to-event analysis prevents any firm conclusions regarding treatment persistence or drug survival. The reported switching rate should therefore be interpreted as a crude descriptive measure and not as a valid proxy for treatment retention. These limitations restrict the interpretation of treatment durability and preclude direct comparison with studies reporting Kaplan–Meier-based retention outcomes.

In addition, treatment line analysis could not be systematically performed due to the limited sample size and retrospective nature of the data. Furthermore, retention rates for individual biologic agents or drug classes could not be assessed due to incomplete time-to-event data and lack of consistent follow-up. Treatment duration analysis focused on adalimumab, as it represented the uniform first-line biologic therapy across all patients, providing a consistent reference point for analysis.

Additionally, standardized efficacy outcomes (such as PASI75 or PASI90) and longitudinal treatment responses were not systematically evaluated. Therefore, the findings should be interpreted with caution and considered hypothesis-generating rather than confirmatory.

Future prospective multicenter studies with larger patient populations are needed to better identify predictors of treatment response, optimize biologic selection, and further evaluate long-term safety in elderly patients with psoriasis. Particular attention should be given to patients aged ≥ 75 years, who remain underrepresented in current research.

It should be noted that psoriasis significantly affects patients’ quality of life, including physical symptoms, psychological burden, and social functioning [23]. In elderly patients, this impact may be amplified by the presence of multiple comorbidities and age-related limitations. In this context, the use of effective and well-tolerated biologic therapy is particularly important, as it may contribute not only to disease control but also to improved quality of life and overall well-being. Overall, these findings contribute to the growing evidence supporting the safe and effective use of biologic therapies in elderly patients with psoriasis in real-world clinical settings.

## 5. Conclusions

This study provides real-world, descriptive insights into biologic therapy use in elderly patients with moderate-to-severe psoriasis. Biologic therapy in this cohort appeared to be generally well tolerated in this cohort, and treatment switching was primarily driven by loss of efficacy rather than comorbidities.

However, given the study limitations, including the small sample size, retrospective single-center design, lack of multivariable analysis, and absence of time-to-event and standardized efficacy assessments, these findings should be interpreted with caution.

Overall, the results should be considered exploratory and hypothesis-generating. Further prospective, multicenter studies with larger patient populations and more comprehensive outcome assessment are needed to confirm these observations.

## Figures and Tables

**Table 1:** Baseline characteristics of elderly psoriasis patients receiving biologic therapy.

Variable	Total (n = 38)
Age, mean ± SD ^1^	70.7 ± 4.7
Age ≥ 75 years, n (%)	8 (21.1%)
Female, n (%)	21 (55.3%)
Male, n (%)	17 (44.7%)
PASI, mean ± SD	19.3 ± 10.2
BSA, mean ± SD	15.0 ± 19.1
Psoriatic arthritis, n (%)	15 (39.5%)
Nail involvement, n (%)	12 (31.6%)
Palmoplantar psoriasis, n (%)	5 (13.2%)
Erythroderma, n (%)	2 (5.3%)
Inverse psoriasis, n (%)	2 (5.3%)
Genital involvement, n (%)	1 (2.6%)
Methotrexate use	33 (86.8%)
MTX inefficacy	18 (47.4%)
MTX adverse events	15 (39.5%)
MTX contraindications	5 (13.2%)
Arterial hypertension	15 (39.5%)
Dyslipidemia	9 (23.7%)
Diabetes	7 (18.4%)
Hepatic steatosis	12 (31.6%)
Thyroid disease	16 (42.1%)
Cardiovascular disease	6 (15.8%)
Lung disease	6 (15.8%)
Kidney disease	2 (5.3%)
Malignancy	6 (15.8%)
Obesity	2 (5.3%)
Infection history	4 (10.5%)
Latent tuberculosis	2 (5.3%)
Number of comorbidities, mean ± SD	2.3 ± 1.5

^1^ Values are presented as mean ± standard deviation (SD) or number (%).

**Table 2:** Biologic therapy and switching.

Variable	n (%)
Switchers ^1^	15 (39.5%)
Non-switchers	23 (60.5%)
Switch due to loss of efficacy	13 (86.7%)
Switch due to adverse events	2 (13.3%)
Second biologic: Guselkumab	6 (40%)
Second biologic: Risankizumab	6 (40%)
Second biologic: Ustekinumab	2 (13.3%)
Second biologic: Secukinumab	1 (6.7%)
Third biologic: Secukinumab	2 (100%)

^1^ Values are presented as number (%).

**Table 3:** Factors associated with biologic switching (univariate analysis).

Variable	No Switch (n = 23)	Switch (n =15)	*p*-Value
PASI, mean ± SD ^1^	17.3 ± 8.4	22.3 ± 12.1	0.139
Psoriatic arthritis, n (%)	13 (56.5%)	2 (13.3%)	0.008
Thyroid disease, n (%)	9 (39.1%)	7 (46.7%)	0.646
Arterial hypertension, n (%)	8 (34.8%)	7 (46.7%)	0.464
Hepatic steatosis, n (%)	7 (30.4%)	5 (33.3%)	0.851
Dyslipidemia, n (%)	5 (21.7%)	4 (26.7%)	0.727
Number of comorbidities, mean ± SD	2.26 ± 1.39	2.33 ± 1.76	0.888

^1^ Values are presented as mean ± standard deviation (SD) or number (%). *p*-values were calculated using the independent-samples t-test and chi-square or Fisher’s exact test, as appropriate.

## Data Availability

The raw data supporting the conclusions of this article will be made available by the authors on request.

## Abbreviations

The following abbreviations are used in this manuscript:

ANA	Antinuclear Antibody
BMI	Body Mass Index
BSA	Body Surface Area
COPD	Chronic Obstructive Pulmonary Disease
HBV	Hepatitis B Virus
HCV	Hepatitis C Virus
ICH	International Council for Harmonisation
IL	Interleukin
IL-17	Interleukin-17
IL-23	Interleukin-23
PASI	Psoriasis Area and Severity Index
SD	Standard Deviation
TI-RADS	Thyroid Imaging Reporting and Data System
WHO	World Health Organization
